# Antidiabetic Effect of Fresh Nopal (*Opuntia ficus-indica*) in Low-Dose Streptozotocin-Induced Diabetic Rats Fed a High-Fat Diet

**DOI:** 10.1155/2017/4380721

**Published:** 2017-02-20

**Authors:** Seung Hwan Hwang, Il-Jun Kang, Soon Sung Lim

**Affiliations:** ^1^Department of Food Science and Nutrition, Hallym University, 1 Hallymdeahak-gil, Chuncheon 24252, Republic of Korea; ^2^Institute of Korean Nutrition, Hallym University, 1 Hallymdeahak-gil, Chuncheon 24252, Republic of Korea; ^3^Center for Efficacy Assessment and Development of Functional Foods and Drugs, Hallym University, 1 Hallymdeahak-gil, Chuncheon 24252, Republic of Korea

## Abstract

The objective of the present study was to evaluate *α*-glucosidase inhibitory and antidiabetic effects of Nopal water extract (NPWE) and Nopal dry power (NADP) in low-dose streptozotocin- (STZ-) induced diabetic rats fed a high-fat diet (HFD). The type 2 diabetic rat model was induced by HFD and low-dose STZ. The rats were divided into four groups as follows: (1) nondiabetic rats fed a regular diet (RD-Control); (2) low-dose STZ-induced diabetic rats fed HFD (HF-STZ-Control); (3) low-dose STZ-induced diabetic rats fed HFD and supplemented with NPWE (100 mg/kg body weight, HF-STZ-NPWE); and (4) low-dose STZ-induced diabetic rats fed HFD and supplemented with comparison medication (rosiglitazone, 10 mg/kg, body weight, HF-STZ-Rosiglitazone). In results, NPWE and NADP had IC_50_ values of 67.33 and 86.68 *μ*g/mL, both of which exhibit inhibitory activities but lower than that of acarbose (38.05 *μ*g/mL) while NPWE group significantly decreased blood glucose levels compared to control and NPDP group on glucose tolerance in the high-fat diet fed rats model (*P* < 0.05). Also, the blood glucose levels of HR-STZ-NPWE group were significantly lower (*P* < 0.05) than HR-STZ-Control group on low-dose STZ-induced diabetic rats fed HFD. Based on these findings, we suggested that NPWE could be considered for the prevention and/or treatment of blood glucose and a potential use as a dietary supplement.

## 1. Introduction

Diabetes is a disease that seriously threatens human health, with more than 200 million people suffering from the disease worldwide. The World Health Organization estimates that the number of patients with diabetes will exceed 360 million by 2030 [1, 2]. Although the current prevalence rates of diabetes in Korea differ depending on region and research, the prevalence rate is around 10% and has shown a 10-fold increase from the prevalence rate of 20 years ago. Treatment methods of diabetes include diet therapy, exercise therapy together with pharmacotherapy, and, clinically, administration of *α*-glucosidase inhibitors, insulin, sulphonylurea, biguanide, and troglitazone. However, fatal side effects such as hypoglycemia and lactic acidosis have been reported with use of medicinal treatments [3]. Because of the tendency to maintain normal blood glucose levels through folk remedies and natural food, rather than with medicinal treatments that incur toxic effects and tolerance, many researchers are actively conducting studies to develop substances from natural materials and food ingredients that can reduce blood glucose [4–6].

Nopal (NP, plant genus* Opuntia*, species* Opuntia ficus-indica*) stems have been traditionally used in Mexico for the treatment of diabetes. Genus* Opuntia* is widely known for its production of mucus, which is a part of dietary fiber and a component of fruits, vegetables, grains, nuts, and beans that humans cannot generally digest. Nopalitos, or soft stems of NP, are eaten in Mexico. To this effect, Frati-Munari et al. studied different aspects of the hypoglycemic effect of* Opuntia *sp. stems. The results of this study suggested that cactus pads reduce absorption of water-soluble dietary fiber content by interrupting absorption of glucose in the intestine and showed reduced blood glucose levels after ingestion of* Opuntia ficus-indica*, with this reduction reaching a statistically significant level after 120–180 min [7]. Trejo-González et al. assessed the blood glucose-reducing activity of purified extract from prickly pear cactus (*Opuntia *sp.) in streptozotocin- (STZ-) induced diabetic mice [8]. They reported that although the mechanism of action is not known, the major substance that reduces blood glucose is presumed to be the dietary fiber in* Opuntia* extract (1 mg/kg body weight/d).

Dietary fiber is classified into water-soluble dietary fiber and non-water-soluble dietary fiber. Water-soluble dietary fiber is composed of mucus, gum, pectin, and hemicelluloses, while non-water-soluble dietary fiber is composed of cellulose, lignin, and a large hemicellulose fraction. The gel in the three-dimensional structure formed by water-soluble dietary fiber is known to prolong the passage of food through the intestine.

However, most preceding studies used samples from NP which was dried and powdered by the sun. Therefore, the purpose in our study was to investigate the evaluation for the prevention and treatment of antidiabetic effect from water extracts of fresh NP stem on the blood glucose control effect by conducting glucose tolerance experiments in the STZ-induced diabetic animal model fed a high-fat diet, as well as the *α*-glucosidase activity in vitro and dietary fiber contents.

## 2. Materials and Methods

### 2.1. Chemicals and Reagents

Streptozotocin (STZ), acarbose, *α*-glucosidase,* p*-nitrophenyl-*α*-D-glucopyranoside, and sodium carbonate were purchased from Sigma-Aldrich. All other chemicals and solvents, unless otherwise specified, were guaranteed reagent grade and purchased from Sigma-Aldrich Chemical Co.

### 2.2. Plant Materials

Plant material used in this study was dried* Opuntia ficus-indica *stem (for which the general name is NP) collected in the Puebla State of Mexico (April 2009). The plants were identified by Emeritus Professor H. J. Chi at Seoul National University, and voucher specimens were deposited in the Center for Efficacy Assessment and Development of Functional Foods and Drugs, Hallym University, with voucher number RIC-2009-NP-0815. Collected dried powder (NPDP) was ground into ultrafine particles, and stems and distilled water were put into an ultrahigh-speed low-temperature vacuum extractor, which was maintained at 80–90°C for 3 h to produce water extract powder of fresh NP stem (NPWE). Extract was filtered, distilled, and put into a washed container, which was then frozen in a deep freezer set to −70°C, put into a freeze dryer, and dried.

### 2.3. Insoluble Dietary Fiber Content (IDF)

Insoluble dietary fiber was quantified according to the method of Prosky et al. [9, 10] as follows: 0.5 g each of NPWE and NPDP were put into separate 500 mL beakers. Then, 25 mL of phosphate buffer (pH 6.0) was added to each beaker to adjust pH to 6 ± 0.2, and 50 *μ*L of Termamyl 120 L solution (120,000 units/mL, NOVO Industri A/S, Copenhagen, Denmark) was added. Then, the beakers were heated to 95°C in a water bath, shaken for 30 min in 5-minute intervals, cooled to room temperature, and adjusted to pH 7.5 ± 0.2 by adding 0.275 N NaOH solution. Then, 2.5 mg of protease was added, and the reaction was allowed to proceed for 30 min at 60°C, with the beakers being shaken at 5-minute intervals. Solutions were cooled to room temperature and adjusted to pH 4.5 ± 0.2 by adding 0.325 M HCl solution. Amyloglucosidase solution (0.15 mL) was added. Beakers were covered with aluminum foil and the reaction was allowed to proceed for 30 min at 60°C, with the beakers being shaken at 5-minute intervals. After the contents were decomposed by enzymes through a filtering crucible with earned constant weight which includes celite, leftover sediments in the crucible were vacuum-filtered, washed in the order of 100 mL of water, 10 mL of 95% ethanol, and 10 mL of acetone, dried in a 70°C vacuum oven overnight, moved to a desiccator to cool for 15 min, and weighed. The protein content of one of two samples was measured using the micro-Kjeldahl method [11], and a nitrogen factor of 6.25 was applied. In addition, the ash content of the other sample was determined with the following procedure: the sample was ashed for 5 h in 525°C, and the ashing furnace was turned off and left overnight and at 130°C for 2. The sample was cooled down in desiccators for 15 min, and ash content was weighed.

### 2.4. Soluble Dietary Fiber Content (SDF)

Ten milliliters of filtrate and water with washed sediments was acquired from the process of measuring insoluble dietary fiber content, adjusted to 50 g with water, and moved to a beaker, to which 200 mL of 95% ethanol heated to 60°C was added. This was left for 60 min so that sediments could form at room temperature. The sediments of a 1 G3 filtering crucible with constant weight that included celite soaked in 78% celite ethanol were vacuum-filtered and washed in the order of 30 mL of 78% ethanol, 10 mL of 95% ethanol, and 10 mL of acetone and then dried in a 70°C vacuum oven for 12 h. The weights of sediments were acquired by weighing the glass crucible cooled in the desiccators, and ash and protein contents were weighed with the same method as insoluble dietary fiber. The same procedures were used to measure the blank samples.

After the completion of the above process, content of dietary fiber was calculated as follows:

(1) Blank  B=R−P−A,Total  dietary  fiber  %=R−P−A−BM×100,

where *R* is weight of sediments after enzyme treatment, *P* is protein amount, *A* is ash amount, and *M* is weight of sample.

### 2.5. Assay Method of *α*-Glucosidase Inhibitory Activity

Inhibitory activity of *α*-glucosidase was measured using Kim's method [12] with modifications. 2 mL of enzyme-originated 2.1 units melted in PBS and 400 *μ*L of sample were mixed and reacted for 10 min at 37°C, and 1 mL of 0.55 mM* p*-nitrophenyl-*α*-D-glucopyranoside was added and reacted for 10 min at 37°C. Then, the reaction was terminated by adding 1.6 mL of 0.1 M Na_2_CO_3_, and inhibition rate was calculated by measuring absorbance at 405 nm:

(2) $$\begin{array}{c} \text{Inhibition rate }\left(\;\%\;\right)=\left\{\;1-\left(\;\frac{\text{Sample O.D.}}{\text{Control O.D.}}\;\right)\;\right\}\times 100, \end{array}$$

where Control O.D. is absorbance of test liquid with PBS applied instead of sample and Sample O.D. is absorbance of test liquid with sample applied.

### 2.6. Experimental Animals

Experimental animals used in this study were male Sprague Dawley rats with body weight of 180–200 g purchased from Koatech Inc. They were adapted to a breeding environment of 23 ± 1°C, with 60 ± 5% humidity, below 60 phones, less than 20 ppm odor, 150–300 lux illumination, and 12-hour light and shade cycle for one week with sufficient food and water. Experiments with animals, as well as their breeding and management, were conducted in accordance with the “Guide for the Care and Use of Laboratory Animals,” and experiments were performed with the authorization of the Ethics Committee of Hallym University (Hallym-2009-77).

### 2.7. Oral Glucose Tolerance Test of NPDP and NPWE in the Fed a High-Fat Diet Rats

Experiments were conducted in order to verify the difference in the blood glucose reduction effect between NPDP and NPWE. Animals adapted to the breeding room were supplied with a high-fat diet for 4 weeks, fasted for 12 h, and separated into the following three groups, with ten animals in each group: a group with no extract injected (control), a group injected with 100 mg/kg NPDP, and a group injected with 100 mg/kg NPWE. Thirty minutes after injection of test samples, 2 g/kg of glucose was orally administered.

### 2.8. Oral Glucose Tolerance Test NPWE in the STZ-Induced Diabetic Rats Fed a High-Fat Diet

After supplying rats with a high-fat diet for 4 weeks, 34 mg/kg STZ (in 0.1 M citrate buffer) was administered by intraperitoneal injection in experimental animals that had been fasted for 12 h. After 1 week, animals with fasting blood glucose of 250 mg/dL were selected for experiments, by measuring blood glucose of blood from the tail vein with a blood glucose monitoring device (Accu-Chek, Germany). Animals were separated into four experimental groups, with ten rats per group. The groups were as follows: a positive control group which was supplied with normal diet with no test samples (RD-Control), a negative control group supplied with a high-fat diet with STZ administration and no test samples (HF-STZ-Control), a group supplied with a high-fat diet with STZ administration and 100 mg/kg of orally administered NPWE (HF-STZ-NPWE), and a group supplied with a high-fat diet with STZ administration and 10 mg/kg of rosiglitazone as comparison medication (HF-STZ-Rosiglitazone). The animal group that was not administered test sample or medication was instead administered carboxymethyl cellulose (CMC, Sigma, USA) solution, which was used in test sample dilution, while each test sample was orally administered at 4 p.m. each day. Test samples and comparison medications were orally administered in all experimental animal groups every day for 4 weeks. Then, the animals were fasted for 12 h, and 2 g/kg of glucose was orally administered.

### 2.9. Oral Glucose Tolerance Test (OGTT) and Biochemical Analysis

At time points of 0 min, 30 min, 1 h, 2 h, and 3 h after glucose administration, blood was collected from the tail vein and blood glucose was measured with a blood glucose monitoring device and blood was collected from the orbital vein of animals fasted for 15 h and contained in SST Vacutainers. Plasma was centrifuged at 3,000 rpm for 15 min and then analyzed with a biochemical measuring instrument for blood index.

### 2.10. Statistical Analysis

Data are expressed as mean values ± SD and comparisons among data were carried out using Student's unpaired *t*-tests or one-way analyses of variance, as appropriate. Mean values were considered significantly different when *P* < 0.05.

## 3. Results and Discussion

### 3.1. Dietary Fiber Content of NPDP and NPWE

Contents of dietary fiber contained in NP stems are presented in Table 1. NPDP contained 4.99% SDF and 53.46% IDF, while NPWE contained only 45.92% SDF, demonstrating that around half of the extract was soluble dietary fiber.

### 3.2. Evaluation of NPDP and NPWE on *α*-Glucosidase Inhibitory Activity In Vitro

The inhibitory activity of NP of *α*-glucosidase is presented in Table 2. NPDP inhibited activity of *α*-glucosidase by 43.59%, 41.50%, 23.52%, and 8.67% at concentrations of 100, 50, 25, and 10 *μ*g/mL, respectively, while NPWE inhibited *α*-glucosidase activity by 53.23%, 47.22%, 44.65%, and 28.89% at concentrations of 100, 50, 25, and 10 *µ*g/mL, respectively. Thus, NPDP and NPWE both showed lower inhibitory activity than that of acarbose, which inhibited *α*-glucosidase activity by 68.39%, 59.41%, 49.34%, and 39.32% at the same concentrations. NPDP showed inhibitory activity with an IC_50_ value of 86.68 *μ*g/mL while NPWE had an IC_50_ value of 67.33 *μ*g/mL, with both exhibiting a lower level of inhibitory activity than the standard medication acarbose (IC_50_ of 38.05 *μ*g/mL).

Although NP had lower inhibitory activity than acarbose (which was used as comparison medication), it was judged that NP has a significant effect in the body even in small amounts. Usually, the intake of the starch is decomposed to monosaccharide by *α*-glucosidase in small intestine and the monosaccharide is absorbed and blood sugar rises. Both NPWE and NPDP showed inhibitory activity on *α*-glucosidase and are expected to prevent radical increase of blood glucose levels as decomposition into monosaccharide by *α*-glucosidase in the small intestine.

Commercially available *α*-glucosidase inhibitors, such as acarbose, may be restricted in their use as they cause side effects such as abdominal distension, nausea, and diarrhea in cases of long-term use. Thus, studies are actively being conducted to find hypoglycemic agents from natural materials with few side effects. It was found that extracts of mulberry leaf, mulberry root, and* Scutellaria* had high levels of inhibitory activity of *α*-glucosidase, and inhibitors of *α*-glucosidase were separated from these extracts [13]. This study also verified the function of NPWE as a hypoglycemic agent by confirming the inhibitory activity of NPWE on *α*-glucosidase.

### 3.3. Effects of NPDP and NPWE on Glucose Tolerance in the Fed a High-Fat Diet Rats

**Figure 1:**
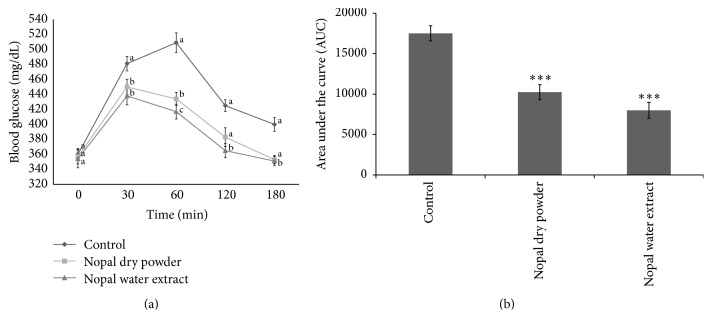
Single-term oral glucose tolerance tests (a) of Nopal dry powder (NPDP) and water extract (NPWE) after 12 h food deprivation in SD rats fed a high-fat diet. (b) Area under the blood glucose concentration curve was measured over 180 min (AUC-180 min). Values are expressed as the mean ± SE (*n* = 10). Different letters in the same time (a) show statistically significant differences, *P* < 0.05. ^*∗∗∗*^*P* < 0.001 versus Control.

Glucose tolerance tests conducted for each experimental group are presented in Figure 1(a). The control group's blood glucose level was maintained in the range of 481 ± 25 mg/dL to 509 ± 9 mg/dL 30 min to 1 h after administration of glucose and in the range of 425 ± 9 mg/dL to 400 ± 8 mg/dL 120 min to 180 min after administration, showing general glucose tolerance. The group administered NPDP and NPWE had glucose levels of 425 ± 9 mg/dL and 400 ± 8 mg/dL, respectively, 30 min after administration of glucose. These levels are lower than those in the animal group without any test samples, and blood glucose levels 60 min after administration of glucose were lower than those 30 min after, displaying distinct differences from the control group. Animal groups administered NPDP and NPWE returned to the initial blood glucose level 180 min after administration and the blood glucose levels of NPWE group were significantly decreased in comparison to control group (*P* < 0.05). And significant difference was shown in the over 30 min zone between NADP and NPWE. When the AUC was compared between groups, the NPWE group significantly showed 45.67% reduction (*P* < 0.001) than the control group (Figure 1(b)). Judging from the fact that animals administered NPWE had lower blood glucose levels in overall time slots, it was suggested that NPWE improved glucose tolerance better than NPDP. Based on this result, NPWE was used for OGTT in the STZ-induced diabetic rats fed a high-fat diet.

### 3.4. Effect of NPWE on Glucose Tolerance in the STZ-Induced Diabetic Rats Fed a High-Fat Diet

**Figure 2:**
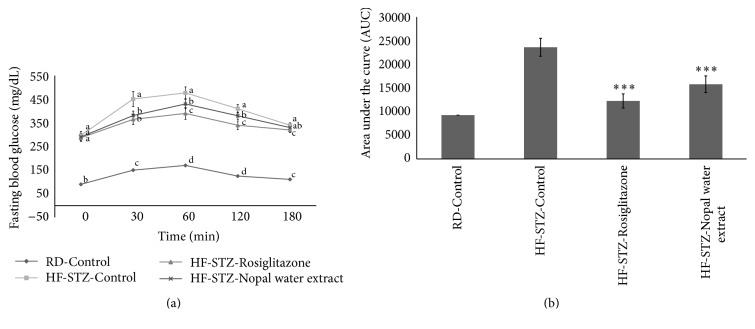
Long-term oral glucose tolerance tests (a) of Nopal water extract (NPWE) after 12 h food deprivation in STZ-induced SD rats fed a high-fat diet. (b) Area under the blood glucose concentration curve was measured over 180 min (AUC-180 min). Values are expressed as the mean ± SE (*n* = 10). Different letters in the same time (a) show statistically significant differences, *P* < 0.05. ^*∗∗∗*^*P* < 0.001 versus HF-STZ-Control.

As shown in Figure 2(a), the blood glucose levels of HR-STZ-NPWE and HR-STZ-Rosiglitazone groups were significantly decreased (*P* < 0.05) compared to HR-STZ-Control group. The fasting blood glucose concentration of HR-STZ-Control group (297.81 mg/dL) was significantly higher than that of the RD-Control group (90.17 mg/dL) at 0 min. Blood glucose level of HF-STZ-Control group was 477.10 mg/dL when the blood glucose of the experimental animals reached its maximum value after the administration of starch at 60 min. However, blood glucose levels were decreased to 430.23 mg/dL in HF-STZ-NPWE after the administration of starch at 60 min. In case of HF-STZ-Control group, after the administration of starch, blood glucose levels increased to 451.17 and 477.10 mg/dL at 30 and 60 min, respectively, while level of blood glucose of HF-STZ-NPWE group decreased significantly, which were 381.87 and 430.23 mg/dL over the same time period. After treatment with NAWP (100 mg/kg), the fasting blood glucose levels were significantly lower (*P* < 0.05) than that of the HF-STZ-Control group at time 30 (19.0%), 60 (18.24%), and 120 (7.31%) min, respectively. HR-STZ-Rosiglitazone showed changes in the blood glucose of 365.02 and 390.71 mg/dL at 30 and 60 min, respectively, and this group showed a marked effect on blood glucose reduction. In addition, when the AUC was compared between groups, the HF-STZ-Rosiglitazone and HF-STZ-NPWE showed 48.09% and 33.10% reductions, respectively (*P* < 0.001), compared to the HF-STZ-Control group (Figure 2(b)).

### 3.5. Analysis of Blood Indices

Blood indices for each experimental group were analyzed, and results of analysis are presented in Table 3. Although HF-STZ-NPWE group was significant difference in other items among animal groups, levels of aspartate aminotransferase (AST), alanine aminotransferase (ALT), cholesterol, LDL-cholesterol, and triglyceride were significantly decreased (*P* < 0.05) and total protein was increased. However HDL-cholesterol was not significantly increased. AST was the highest in HF-STZ-Control, with levels of 133.60 mg/dL, and the comparison HF-STZ-Rosiglitazone showed AST levels of 114.80 mg/dL. Although the HF-STZ-NPWE did not reach the level of healthy animals with 111.11 mg/dL, a significantly low level was observed on AST. The negative control group showed a level of ALT of 91.40 mg/dL, the RD-Control showed a level of 51.60 mg/dL, and HF-STZ-NPWE was significantly lower with 66.64 mg/dL lower than HF-STZ-Control. On the other hand, the HF-STZ-Rosiglitazone had ALT levels of 113.9 mg/dL (*P* < 0.05).

The HDL-cholesterol level of the RD-Control was 54.70 mg/dL and that of the HF-STZ-Control was 56.60 mg/dL, while the HF-STZ-NPWE showed a level of 49.17 mg/dL, which is close to the normal level. Even though HF-STZ-Rosiglitazone showed ALT levels of 113.90 mg/dL, this was still higher than the level of ALT of HF-STZ-NPWE. The blood creatine concentration was high, but no significant difference was observed.

The creatine is measured as indicators reflecting the status of renal function prior to diabetic nephropathy, with creatine being a substance that is generated as a result of the creatine phosphate metabolism in the muscle. Under normal conditions, creatine is isolated from the muscle at a relatively constant speed, so the blood creatine concentration is constant when filtered through the glomerulus, and is neither reabsorbed nor metabolized. In a chronic kidney disease due to diabetes, the glomerular filtration rate decreases and the concentration of blood creatine increases. In the present study, the creatine concentration tended to increase in the diabetes-induced group, but there was no significant difference. The degradation of kidney function can be assumed, but no serious complications appeared.

While the content of the soluble dietary fiber in the NPDP test sample was 4.99%, the content increased to 45.92% as a result of water extraction. On the contrary, content of insoluble dietary fiber was eliminated through the water extraction process. The results of the *α*-glucosidase inhibition activity experiment showed that NPWE had a slightly lower level of inhibitory activity, with an IC_50_  of 67.33 *μ*g/mL, than the comparison medicine acarbose. Recognizing the blood glucose-decreasing effect of NPWE through short-term OGTT, this study did an OGTT in the STZ-induced diabetic rats fed a high-fat diet with NPWE (HF-STZ-NPWE). In STZ-induced diabetic animals, HF-STZ-NPWE showed lower blood glucose levels than the diabetic comparison group, and a blood component test also confirmed the normalization effect of cholesterol in the blood.

NP and dietary fibers have been previously reported on the antihyperglycemic effect included in STZ-induced diabetic rats [14], type II diabetes mellitus patients [5], noninsulin-dependent diabetes mellitus patients [7], and serum lipids, glycemia, and body weight [15]. In addition, previous investigations into the antidiabetic effects of dietary fiber on the adsorption of glucose by Ou et al. reported that postprandial serum glucose was lowered by various dietary fibers including xanthan gum, carboxymethyl cellulose, guar gum, and water-soluble dietary fiber from wheat bran [16]. For these reasons, three pathways were suggested that first is to increase the viscosity of the small intestinal content and retard the diffusion of glucose. The second is to adsorb glucose and prevent its diffusion and, finally, to inhibit the activity of *α*-glucosidase and postpone the release of glucose from starch by Ou et al. [16]. In addition, Munoz et al. reported that dietary fiber sources corn bran (CB), soy hulls (SH), freeze-dried apple powder (AP), and freeze-dried carrot powder (CP) were fed to 15 men as part of a mixed diet containing fiber sources 92.1% (CB), 86.7% (SH), 25.6% (AP), and 31.0% (CP) fiber. The result was that 15 men showed significantly improved oral glucose tolerance by these dietary fiber sources [17].

In the prevention and treatment of diabetes, it is important to control blood glucose after eating. Hyperglycemia after feeding is known to be a symptom not only of severe diabetes, but also of slight diabetes, in which high glucose levels on an empty stomach are not a symptom [18]. Hyperglycemia after eating has been reported to worsen the diabetic condition and cause macrovascular and microvascular complications by decreasing insulin sensitivity and lowering insulin secretion [19]. In addition, AST and ALT are used as indices of liver damage, as their concentration increases in the blood when liver cells are damaged [20]. ALT activity increased in the control group given no medication. Moreover, administration of medication and NPWE tends to reduce activity of AST (16.8%), ALT (27.0%), and cholesterol (24.4%) more than the HF-STZ-Control group (Table 3). Studies by Young and Stout [21] and Bursch et al. [22, 23] reported that plasma cholesterol content of diabetic white rats showed a similar level to that of normal rats and presumed that total cholesterol in all induced diabetic groups increased as free fatty acid, not carbohydrates, was used as an energy source in the metabolism of diabetic rats, during which cholesterol is synthesized [24, 25]. They maintained that, in induced diabetic animals, increases in neutral lipids and decreases in HDL-cholesterol are observed [26] and that high cholesterol blood levels are caused by an increase of cholesterol synthesis in the body [27]. This phenomenon takes place in insulin-dependent or insulin-independent diabetics and is heavily influenced by dietary components; among them, dietary fiber is known to reduce cholesterol content by absorbing cholesterol or bile acid and excreting it in the feces, using cholesterol in the synthesis of bile acid [28].

Following the suggestion that deficiency of dietary fiber may be a cause of diabetes, Lee et al. reported that a high carbohydrate, high fiber diet decreases blood glucose and insulin requirements in insulin-dependent or insulin-independent diabetics and lowers concentrations of lipids in the blood [29]. The beneficial effects of dietary fiber have been supported by numerous studies, and there is a consensus of opinions that the focus in dietary therapy for diabetic patients should be on a high complex carbohydrate, high dietary fiber, and low fat diet.

Our study has several strengths. First, we analyzed concentration of dietary fiber in NP stem extract used as dietary supplements. We fed animals with a high-fat diet that might cause prediabetes condition in humans supplemented with NPWE, allowing us to calculate intake of dietary fiber from food intake. Third, in an experimental approach we have adapted, diabetes is induced by a single intraperitoneal STZ injection in citrate buffer in an amount of 15–90 mg/kg of animal body weight. This model has been commonly used to study the pathophysiology for humans with type 2 diabetes model. However, our study has some limitations. First, we did not perform morphological examination to further substantiate the beneficial effect of NPWE on pancreatic *β*-cell function and gut to reduce or inhibit intestinal glucose digestion and absorption. Second, we did not analyze other bioactive components, such as polyphenols and flavonoids. For this reason, we suggest that bioactive components in NPWE should be identified too on antiglycemic effect. Further studies focusing on the bioactive components are needed to fully understand the functional mechanism of NPWE and this study showed that dietary supplementation with NP could potentially contribute to nutritional strategies for the prevention and treatment of diabetes mellitus.

## 4. Conclusion

In conclusion, the study demonstrates that NPWE significantly improves deranged carbohydrate metabolism in STZ-induced diabetic rats fed a high-fat diet. The results suggest that, at least in part, NPWE preparations can be used as a nutraceutical agent to ameliorate diabetes type 2 and are necessary to further elucidate the antidiabetic of action of NPWE.

## Figures and Tables

**Table 1:** Concentration of soluble dietary fiber (SDF) and insoluble dietary fiber (IDF) in Nopal dry powder (NPDP) and water extract (NPWE).

Dietary fibers	Nopal dry powder (%)	Nopal water extract (%)
SDF^(1)^	4.99 ± 0.42	45.92 ± 5.17
IDF^(2)^	53.46 ± 4.91	ND^(3)^

__^(1)^SDF: soluble dietary fiber

__^(2)^IDF: insoluble dietary fiber

__^(3)^ND: not detected.

**Table 2:** Inhibitory effect of *α*-glucosidase activity of Nopal dry powder (NPDP) and water extract (NPWE).

Extracts	Concentration (*μ*g/mL)	Inhibition (%)	IC_50_ (*μ*g/mL)^(1)^
Nopal dry powder	100	43.59	86.68 ± 9.97
	50	41.50	
	25	23.52	
	10	8.67	

Nopal water extract	100	53.23	67.33 ± 6.47
	50	47.22	
	25	44.65	
	10	28.89	
	5	19.02	
	2.5	5.12	

Acarbose^(2)^	100	68.39	38.05 ± 2.80
	50	59.41	
	25	49.34	
	10	39.32	
	5	34.41	
	2.5	16.46	

__^(1)^The IC_50_ value was defined as the half-maximal inhibitory concentration and mean of 3 duplication analyses of each sample.

__^(2)^Acarbose was positive control.

**Table 3:** Analytical methods of blood plasma chemistry items.

Biomarker	Raw diet (mg/dL)	High fat diet-STZ (mg/dL)		
	RD-Control	HF-STZ-Control	HF-STZ-NPWE	HF-STZ-Rosiglitazone
Albumin	3.20 ± 0.04^a^	2.66 ± 0.05^ab^	2.87 ± 0.03^ab^	2.95 ± 0.13^a^
Total protein	6.71 ± 0.11^a^	4.93 ± 0.12^d^	5.78 ± 0.12^c^	6.25 ± 0.31^b^
AST^(1)^	82.80 ± 4.50^c^	133.60 ± 5.92^a^	111.11 ± 9.69^b^	114.80 ± 11.60^b^
ALT^(2)^	51.60 ± 0.84^d^	91.40 ± 2.1^b^	66.64 ± 3.48^c^	113.90 ± 4.50^a^
Cholesterol	62.30 ± 5.47^c^	83.50 ± 1.01^a^	63.06 ± 9.97^c^	75.80 ± 6.47^b^
Triglyceride	109.30 ± 8.01^a^	158.30 ± 4.78^a^	132.29 ± 9.57^a^	143.40 ± 3.04^a^
HDL-cholesterol	54.70 ± 5.17^a^	56.60 ± 3.21^a^	49.17 ± 4.91^b^	39.90 ± 2.80^c^
LDL-cholesterol	7.97 ± 1.28^b^	12.33 ± 1.12^a^	3.60 ± 0.83^c^	2.00 ± 1.00^d^
Creatine	0.54 ± 0.01^b^	0.55 ± 0.03^b^	0.63 ± 0.02^a^	0.59 ± 0.01^ab^
Uric acid	0.25 ± 0.03^d^	0.38 ± 0.05^c^	0.77 ± 0.17^a^	0.68 ± 0.02^b^

__^(1)^AST: aspartate aminotransferase

__^(2)^ALT: alanine aminotransferase

Values are expressed as the mean ± SE (*n* = 10). Different letters in rows show statistically significant differences, *P* < 0.05.
